# Perry Disease: Bench to Bedside Circulation and a Team Approach

**DOI:** 10.3390/biomedicines12010113

**Published:** 2024-01-05

**Authors:** Takayasu Mishima, Junichi Yuasa-Kawada, Shinsuke Fujioka, Yoshio Tsuboi

**Affiliations:** Department of Neurology, Fukuoka University, Fukuoka 814-0180, Japan; mishima1006@fukuoka-u.ac.jp (T.M.); jkawada@fukuoka-u.ac.jp (J.Y.-K.); shinsuke@cis.fukuoka-u.ac.jp (S.F.)

**Keywords:** Perry syndrome, rare disease, genetic testing, pathogenesis, common disease, research, treatment, bedside, technological applications, team

## Abstract

With technological applications, especially in genetic testing, new diseases have been discovered and new disease concepts have been proposed in recent years; however, the pathogenesis and treatment of these rare diseases are not as well established as those of common diseases. To demonstrate the importance of rare disease research, in this paper we focus on our research topic, Perry disease (Perry syndrome). Perry disease is a rare autosomal dominant neurodegenerative disorder clinically characterized by parkinsonism, depression/apathy, weight loss, and respiratory symptoms including central hypoventilation and central sleep apnea. The pathological classification of Perry disease falls under TAR DNA-binding protein 43 (TDP-43) proteinopathies. Patients with Perry disease exhibit *DCTN1* mutations, which is the causative gene for the disease; they also show relatively uniform pathological and clinical features. This review summarizes recent findings regarding Perry disease from both basic and clinical perspectives. In addition, we describe technological innovations and outline future challenges and treatment prospects. We discuss the expansion of research from rare diseases to common diseases and the importance of collaboration between clinicians and researchers. Here, we highlight the importance of researching rare diseases as it contributes to a deeper understanding of more common diseases, thereby opening up new avenues for scientific exploration.

## 1. Introduction

The low incidence of rare diseases influences patient resources, diagnostic and therapeutic approaches, and research on mechanisms and potential treatments [1]. For many rare diseases, collaboration among researchers is often difficult and guidelines do not exist. Against this background, we focus on one rare disease, Perry disease/syndrome, and discuss strategies for research into other rare diseases.

## 2. History of Perry Disease Research

**Figure 1:**
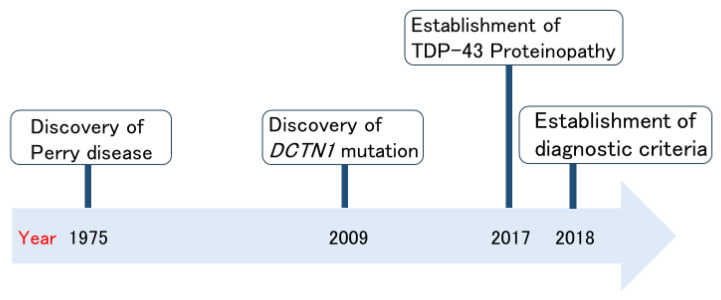
Milestones of Perry disease research.

Perry disease is a neurodegenerative disease; it is rare, autosomal dominant, and patients display the clinical symptoms of parkinsonism, respiratory symptoms, depression/apathy, and weight loss [2,3]. Figure 1 shows the history of Perry disease research. In 1975, Perry disease was initially reported and named by Perry et al. [4] in Canada. The identification of *DCTN1* mutations in 2009 accelerated Perry disease research [5]. Just after the establishment of a new TAR DNA-binding protein 43 (TDP-43) proteinopathy [6,7], in 2018, international diagnostic criteria for Perry disease were created [8]. Perry disease generally produces uniform clinical features and characteristic pathological findings; therefore, in the same year, we proposed a name change from Perry syndrome to Perry disease [8]. Here, we summarize Perry disease research to date from the clinician’s perspective. We also discuss the importance of bench to bedside strategies regarding Perry disease. Based on this understanding, we would like to highlight the often-overlooked value of rare disease research, particularly its potential in contributing to the elucidation of common disease pathogenesis.

## 3. Importance of Early Diagnosis in Perry Disease

All diseases, including rare diseases, benefit from early diagnosis. In this section, we discuss the necessity of early diagnosis using Perry disease as our example. Early diagnosis is always critical during therapeutic intervention, and the publication of international diagnostic criteria enables the early diagnosis of Perry disease [8]. Indeed, prior to the development of the diagnostic criteria, there were only about 20 reported families with Perry disease; however, in the approximately five years since the publication of the diagnostic criteria (see Table 1 [8]), this number has increased to over 30. Parkinsonism, family history, and *DCTN1* mutation are sufficient to diagnose Perry disease, with an emphasis on family history. For early diagnosis, genetic testing is recommended if an individual has parkinsonism and a family history of parkinsonism or respiratory symptoms. This is particularly important given that patients with Perry disease often present before the age of 50 and show significant disease progression, with respiratory failure and sudden death occurring within just 5 years of disease onset. Although the previously reported case involving p.K56R mutation in the *DCTN1* gene with slow progression [9] is important, it did not meet the proposed Perry disease diagnostic criteria due to the lack of family history and should be discussed in future studies [8]. Since Perry disease is often difficult to diagnose in the early stages, key points for clinical features are listed in Table 2.

Next-generation sequencing technologies have now revealed gene mutations in patients without a family history [10,11] and genetic testing is being more widely used in the diagnosis of neurological diseases [12]. Of particular importance are compound heterozygous mutations. Many case reports have been published, and one example shows that novel heterozygous mutations in the *SYNE1* gene have also been reported in juvenile amyotrophic lateral sclerosis (ALS) [13]. Regarding the *DCTN1* gene, a case of parkinsonism with two trans variants has been reported [14], and we predict the diagnosis of more such cases in the future. Therefore, a revision of the diagnostic criteria of Perry disease may be needed. As mentioned above, revisions in diagnostic criteria for all diseases should be ongoing in order to keep pace with technological innovations.

## 4. Pathology of Perry Disease

Neurodegenerative diseases characteristically involve neuronal loss and the progressive degeneration in different parts of the nervous system, and coexist with proteinopathies such as tauopathy, synucleinopathy, and TDP-43 proteinopathy [15,16]. Therefore, pathological analysis is essential for elucidating the pathogenesis of these diseases and for developing treatments.

**Figure 2:**
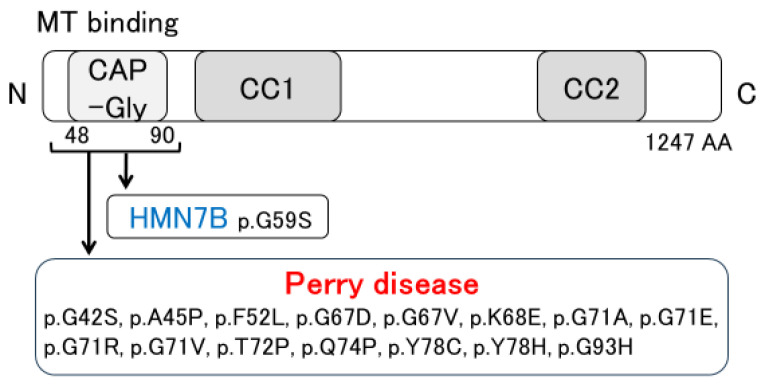
Discovery of *DCTN1* mutations. MT: microtubule; CAP-Gly: cytoskeleton-associated protein glycine-rich; CC: coiled-coil; HMN7B: distal hereditary motor neuropathy 7B.

The pathological features of Perry disease have revealed substantial neuronal loss and gliosis, with few or no Lewy bodies or neurofibrillary tangles present in the substantia nigra [4,6,7,17,18,19,20,21,22,23]. Neuronal loss is detected in the lentiform nucleus, the locus coeruleus, the dorsal raphe nucleus, the periaqueductal gray matter, the hypothalamus, and the brainstem, including putative respiratory neurons in the medulla [6,20,21,24]. In Perry disease patients, TDP-43 pathology is noted in the extrapyramidal system and brainstem [6,7]. Such distribution differs from that of other TDP-43 proteinopathies, such as ALS and frontotemporal lobar degeneration (FTLD) with TDP-43 inclusions (FTLD-TDP) [7]. Furthermore, as described below, our pathological research presents Perry disease as a distinctive type of TDP-43 proteinopathy. Distal hereditary motor neuropathy 7B (HMN7B) is one type of motor neuron disease. As shown in Figure 2, a missense mutation in the *DCTN1* gene also causes HMN7B. The pathology of HMN7B shows no TDP-43 pathology but evidence of dynactin aggregates. Specifically, neuronal cytoplasmic inclusions (NCIs) that are positive for the dynactin subunit p50 (DCTN2) have been observed. Similar inclusions have also been seen in Perry disease [25,26]. Therefore, we set out to evaluate the distribution and morphology of TDP-43 and dynactin pathology in patients with Perry disease, HMN7B, ALS, FTLD-MND, and hippocampal sclerosis (HpScl) in order to clarify Perry disease pathology [7]. Perry disease exhibited a unique TDP-43 pathology, which included abundant NCIs, dystrophic neurites, spheroids, and perivascular astrocytic inclusions. These findings differed from the TDP-43 pathology noted in ALS, FTLD-MND, and HpScl. An immunoelectron microscopic study demonstrated the difference between TDP-43-positive NCIs in Perry disease and those of ALS or FTLD-TDP. In contrast, no TDP-43 pathology was detected in HMN7B. Furthermore, dynactin pathology and p50-positive NCIs remained unseen in ALS, FTLD-MND, and HpScl [7].

Recently, multiple neuropathologies have received much attention [27,28]. A study of aged persons revealed that about 80% of participants had comorbid multiple neuropathologies, including TDP-43 pathology, at autopsy [29]. ALS/parkinsonism dementia complex (ALS/PDC) is also known as a multiple proteinopathy (tauopathy, synucleinopathy, and TDP-43 proteinopathy); however, it remains unclear whether there is a coexistence of proteinopathies [30,31,32,33,34]. Regarding Perry disease, Honda et al. reported that an elderly Perry disease patient harboring a p.F52L mutation with a long-term course had tau and synuclein aggregates at autopsy. This is thought to be due to the possibility that Perry disease exhibits multiple neuropathologies over a long period of time and the effects of aging [35]. Chung et al. also revealed the presence of neurofibrillary tangles in the parahippocampal gyrus during aging in Perry disease [36]. Therefore, analysis of Perry disease pathology may shed light on the study of multiple neuropathologies.

Cryo-electron microscopy (cryo-EM) technology has improved significantly in recent years, making it now possible to determine the atomic coordinates of many biomolecules [37]. New findings in cryo-EM analysis in 2022 from three research groups suggest a potential etiologic commonality of transmembrane protein 106B (TMEM106B) among distinct proteinopathies [38,39,40]. An analysis of the cryo-EM structure of TDP-43 and TMEM106B in patients with Perry disease is needed to further elucidate the pathophysiology. 

In summary, we have updated findings regarding Perry disease pathology. Most of these results were performed by a collaboration between our team and the Mayo Clinic Brain Bank team. We emphasize the importance of gathering samples and data from patients with rare diseases and having these examined by rare disease teams.

## 5. Expansion of *DCTN1* Mutations

Recent advances in clinical and molecular genetics have led to the discovery of many genes responsible for single gene disorders and disease susceptibility genes, and new disease concepts have been proposed. For example, cerebral autosomal dominant arteriopathy with subcortical infarcts and leukoencephalopathy (CADASIL), a typical inherited systemic arterial vessel disease that causes vascular dementia and lacunar infarction, is caused by mutations in the *NOTCH3* gene in an autosomal dominant manner [41,42,43]. Regarding the *NOTCH3* gene, an analysis of 200,000 cases in the U.K. revealed that *NOTCH3* variants are common in the general population and are also susceptibility genes for isolated stroke and vascular dementia [42]. In addition, a recent study conducted in Japan reported that the *RNF213* p.R4810K variant, a susceptibility gene for moyamoya disease, is associated with intracranial arterial stenosis, resulting in atherothrombotic stroke, and impacts endovascular therapy for large vessel occlusion stroke [44,45]. Therefore, the authors proposed the concept of *RNF213*-related vasculopathy [46]. In the area of neurodegenerative diseases, Yabe et al. found changes in the *bassoon* (*BSN*) gene, which translates the BSN in the active zone of presynaptic neurotransmitter release sites, in progressive supranuclear palsy (PSP)-like syndrome patients [47]. They also showed that tauopathy is a novel condition in which tau proteins with three and four repeats accumulate [47]. The *BSN* gene has also been reported to be associated with other neurological disorders, such as multiple sclerosis, and the disease concept of bassoon proteinopathy has been proposed [48].

*DCTN1*, the causative gene for Perry disease, encodes the largest subunit of dynactin (DCTN1/p150^Glued^) [5,49,50,51,52,53,54,55,56]. Dynactin is a multimeric complex that acts as an essential cofactor of the microtubule-based motor cytoplasmic dynein [49,50,51,52,53,54,55,56]. Dynein and dynactin together form the principal motor machinery driving the retrograde transport of cargo within cells [57,58]. DCTN1 is an essential factor in the initiation of dynein-dependent retrograde transport from microtubule plus-ends [59,60]. DCTN1 contains basic domains, a cytoskeleton-associated protein glycine-rich (CAP-Gly) domain, and the coiled-coil 1 (CC1) and CC2 domains. The CC1 and CC2 domains interact with other dynactin subunits and the dynein intermediate chain. The CAP-Gly and basic domains have microtubule binding affinity [60,61,62,63]. Most mutations in Perry disease are located within CAP-Gly domains [8,23,64,65,66,67,68,69,70]. Importantly, a *DCTN1* mutation (p.G59S mutation) causes HMN7B [25,26,71] (Figure 2). Furthermore, *DCTN1* is a risk gene for ALS [72,73,74], and the p.G59R mutation may cause dHMN and ALS [75]. Interestingly, it is reported that a novel p.Q93H mutation in *DCTN1* causes a motor neuron disease phenotype and Perry disease [64]. Wszolek et al. have proposed the disease concept of *DCTN1*-related neurodegeneration [73,74], which is expected to be established along with revised diagnostic criteria.

Genetic counseling, especially predictive genetic counseling, is necessary for the early diagnosis and treatment of hereditary diseases. Since genetic counseling for neurodegenerative diseases began primarily with Huntington’s disease (HD), the HD genetic counseling protocol has been adopted as the standard [76]; however, Perry disease, which is also a severe autosomal dominant disorder and with the aforementioned expansion of the disease concept, may provide a new model for genetic counseling for neurodegenerative diseases.

In this section, we discussed the connection between single gene disorders and common diseases. We predict the expansion of such ideas by many clinicians and researchers. In the future, the frequency of genetic testing is set to increase in clinical settings. Therefore, it is important that genetic counselors are included in clinical teams, as described later.

## 6. Basic Research of Perry Disease

In genetic diseases, the discovery of genetic mutations is essential to the development of research. Next, disease models are required. The reproduction of aggregates is important in neurodegenerative diseases. Regarding Perry disease, dynactin and TDP-43 are targets, and many studies have reproduced dynactin aggregates in cultured cells [77,78,79,80,81]. Our research revealed that truncated mutant forms of DCTN1 cause TDP-43 mislocalization and aggregates. Furthermore, induced pluripotent stem cells (iPSCs) were generated from a patient with Perry disease (p.F52L mutation) and they were differentiated into tyrosine hydroxylase (TH)-positive neurons; dynactin aggregates were identified in the cytoplasm of the patient’s TH-positive neurons, and this partially recapitulated Perry disease pathology [79]. Using biochemical analysis with a panel of truncated mutants, we also found that DCTN1 binds to TDP-43 and demonstrated that the DCTN1 CAP-Gly-basic supradomain, dynactin domain, and C-terminal region interacted with TDP-43, preferentially through its C-terminal region [81]. In mouse models, we have focused on the p.G71A mutation, which presents a typical Perry disease phenotype, and generated transgenic (Tg) and knock-in mice [82,83]. DCTN1^G71A^ transgenic mice showed behavioral defects, parallel apathy-like symptoms, and parkinsonism [82]; furthermore, these knock-in mice displayed a decrease in TH immunoreactivity in the neurons of the substantia nigra [83]. Yu J et al. generated *DCTN1* conditional knockout mice by deleting *DCTN1* in midbrain dopaminergic neurons [84,85]. These mice showed impaired motor coordination and a loss of dopaminergic neurons [85]; however, none of the model mice were able to reproduce the complete clinical phenotypes and pathology of Perry disease patients.

In contrast, the *DCTN1* p.G59S mutation of HMN7B models has also been reported. The p.G59S mutant cells exhibit dynactin aggregates and a reduced binding affinity for microtubules [5,86,87]. Both p.G59S Tg and knock-in mice exhibited the phenotype of motor neuron disease [88,89,90]. Furthermore, the HMN7B mutation was found to disrupt transport within murine primary dorsal root ganglion (DRG) neurons [62,91]. In Perry disease mutations, reductions in microtubule binding affinity for microtubules, dynactin aggregates, autophagic insufficiency, and apoptotic changes were noted in non-neuronal cells as well as the HMN7B mutant [5,77,78,79,80,87]. In the motor neurons of *Drosophila*, HMN7B mutation showed an accumulation of dynein at synaptic termini; however, this was not found in Perry disease mutants, although the latter disrupted flux from the distal neurite (p.Q74P and p.G71R) and a p.Q74P mutation was impaired in inhibiting microtubule catastrophe in murine primary DRG neurons [62,63,91]. These results suggest the possibility of a greater fragility in dopaminergic neurons than in motor neurons in Perry disease. To address this question, similar platform phenotypic comparisons between Perry disease mutations and the HMN7B mutation, using dopaminergic neurons and/or motor neurons differentiated from iPSCs, will be needed [3].

As for treatment approaches, Hosaka et al. reported that reduced TDP-43 protein levels improved neuronal activities in a *Drosophila* model of Perry disease [92]. This approach may be one option for studying the therapeutic treatment of Perry disease [3]. With the development of genome-editing technologies, genetic therapy is being attempted in several neurodegenerative diseases [93,94,95]. Gene therapy is also expected to be used in Perry disease; however, the time at which intervention should start needs to be discussed. It is impossible to follow the natural history of the disease prior to its onset in many patients because Perry disease is rare. Although it is important to analyze the disease in animal models, a Perry disease model has not yet been established; this is required for the future development of research on other diseases. Tacik et al. reported the implantation of a diaphragmatic pacemaker in a patient with Perry disease as a treatment for respiratory symptoms [96]. In the future, it is expected that diaphragmatic pacemakers will be improved, first in animal studies and then by applying techniques such as deep brain stimulation.

**Figure 3:**
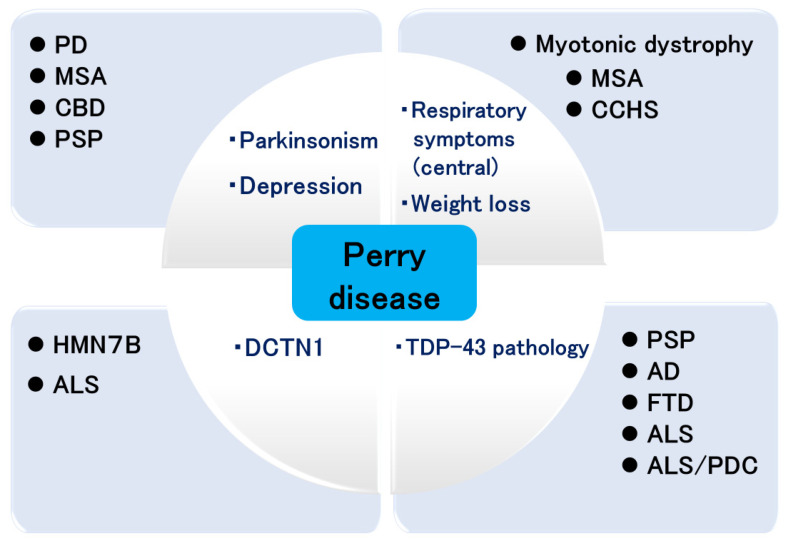
Expansion of Perry disease research. PD: Parkinson’s disease; MSA: multiple system atrophy; CBD: corticobasal degeneration; PSP: progressive supranuclear palsy; HMN7B: distal hereditary motor neuropathy 7B; ALS: amyotrophic lateral sclerosis; CCHS: congenital central hypoventilation syndrome; AD: Alzheimer’s disease; FTD: frontotemporal dementia; PDC: parkinsonism dementia complex.

The attempts in achieving breakthroughs in Perry disease research have led to the study of various diseases (Figure 3). Clinically, such research advances the study of PD and Parkinsonian syndromes, respiratory symptoms, multiple system atrophy, congenital central hypoventilation syndrome [97], and myotonic dystrophy. Genetically, it contributes to the study of HMN7B and ALS, and pathologically it will merge with the studies of other neurodegenerative diseases. 

We have summarized the basic research regarding Perry disease in this section. In rare disease research, findings of common points between rare diseases and common diseases may require collaboration with other researchers. Although Perry disease research is in the process of development, this research trajectory may impact the research of other rare diseases in the future.

## 7. Team Approach for Perry Disease

**Figure 4:**
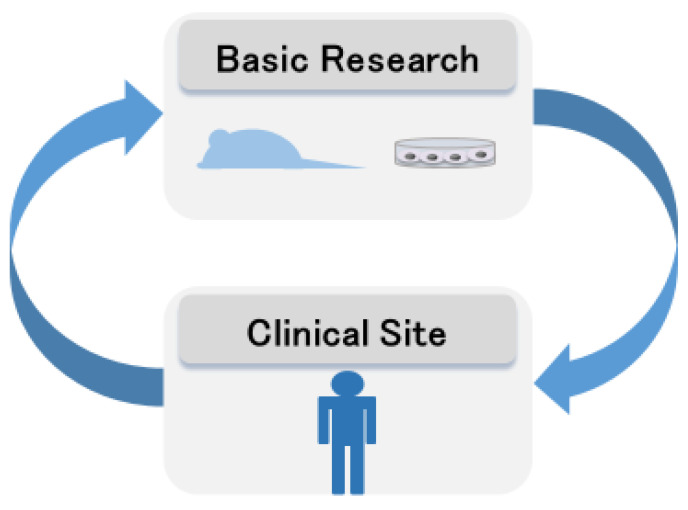
Bench to bedside circulation.

**Figure 5:**
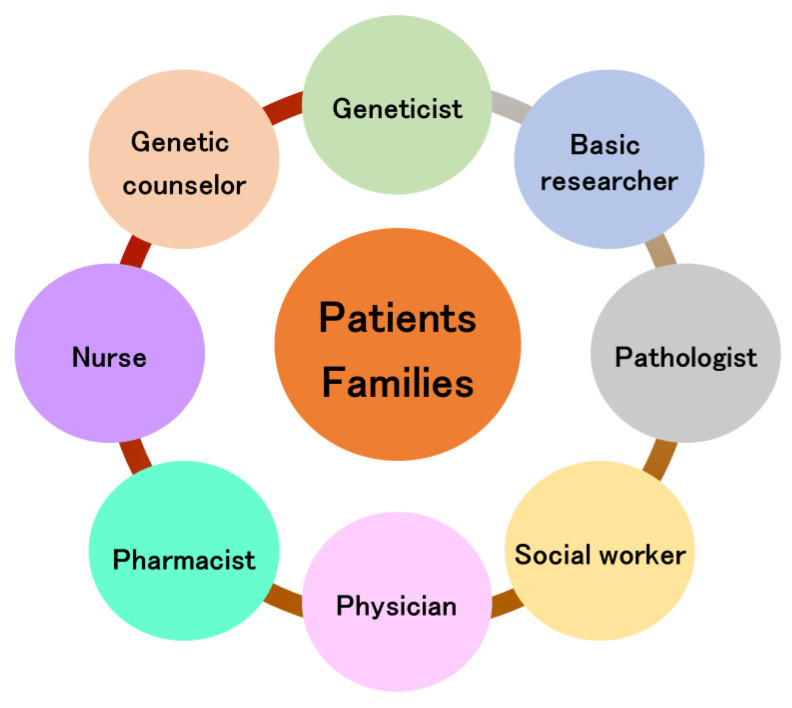
Team approach for rare neurodegenerative diseases.

The importance of the bench to bedside concept has been described in many research fields [98,99,100]. In particular, Hampton suggests the key clinical breakthrough of bench to bedside and back again [101]. Thus, the circulation of information is needed (Figure 4), and it takes teams of clinicians and basic researchers worldwide to achieve such breakthroughs [102,103]. In the clinical setting, a team approach in device-based therapies is widespread [104,105], and the need for a team approach is recognized in the treatment of Parkinson’s disease [106]. Rare neurodegenerative diseases require the involvement of even more professions. The team approach for rare neurodegenerative diseases is shown in Figure 5. In genetic diseases, the role of genetic counselors is important [76], especially in diagnosis, where the involvement of geneticists and basic researchers is essential in assessing the presence or absence of pathological variants. Although the ACMG guidelines are used to evaluate pathological variants [107], these pathological variants may be evaluated for each disease and should be evaluated in a conference including clinicians and geneticists and basic researchers.

Regarding Perry disease, an international conference in Tokyo, Japan, was held from 22 to 23 February 2011 [8]. This meeting led to international collaborative research on Perry disease and the development of international diagnostic criteria [8]. We have just organized a second international conference on Perry disease in Fukuoka, Japan, bringing together clinicians and basic researchers. At this meeting, a revision of diagnostic criteria and the concept of *DCTN1*-related neurodegeneration was discussed. It is highly desirable to establish an international consortium involving various professions on many rare diseases to make breakthroughs in the future. 

**Figure 6:**
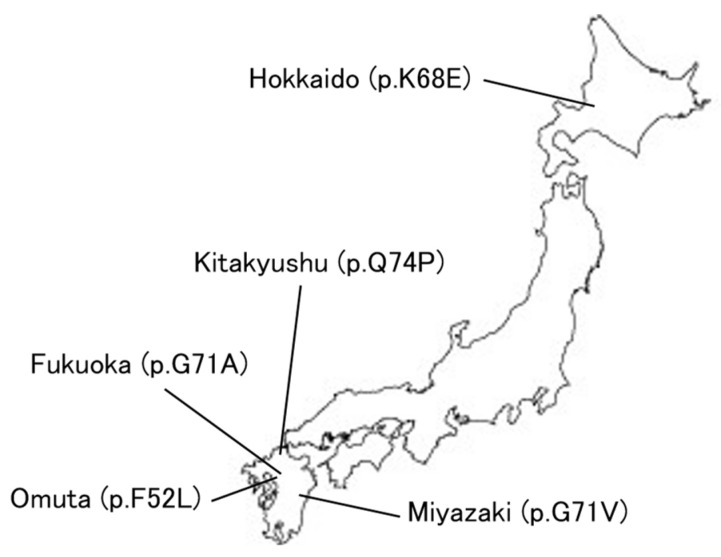
Perry disease in Japan.

COVID-19 has revolutionized medical systems and telemedicine is expanding [106]. Figure 6 shows the location of the current Perry disease families in Japan, and in fact, we are also providing remote genetic counseling in cooperation with other facilities to families of patients with Perry disease who live far away from home. The development of telemedicine may be essential for the treatment of rare diseases and the development of research in this context.

We have discussed a team approach based on Perry disease. It is important that a team approach is consistently implemented from diagnosis through to treatment in clinical settings. For research, a team approach is essential to archive bench to bedside and back again. We expect that team approaches will be seamlessly conducted in the future alongside technological advances.

## 8. Discussion

In the previous sections, we described the history of research regarding Perry disease. It began with Perry, who reported the disease from a family study [4], and the discovery of the *DCTN1* mutation [5], which was the first step toward understanding the pathogenesis of the disease, thanks to international collaboration. Such collaborations led to an elucidation of the pathology [7] and the establishment of the diagnostic criteria of Perry disease [8]. We also described how Perry disease research can guide the study of common diseases through sections on pathology, genetics, and basic research. In the team approach section, we also proposed collaboration on new devices tailored to the social context.

We discussed the importance of research on rare diseases and, needless to say, the study of common diseases helps in the study of rare diseases. Many studies that have been performed on common diseases have not been implemented for rare diseases. This is also true for Perry disease research. Perry disease is rare, so the analysis of its pathogenesis is poor. For example, research into the affected neural circuits and brain regions associated with Perry disease will provide a clearer understanding of the disease’s symptoms. In particular, analyses of specific regions and their role in affecting motor control, respiratory function, and emotional processing could shed light on the neurological basis of the symptoms. In addition, studies of neurodegenerative processes in affected brain regions, such as neuronal loss, gliosis, and protein aggregates, will clarify pathophysiological mechanisms [108,109]. Future research regarding how these processes relate to symptoms and disease progression will strengthen the links between pathophysiology and clinical presentation [110,111].

Several limitations of this review need to be acknowledged. First, this manuscript is a review of only one disease, Perry disease. Second, a treatment for Perry disease has not been established, and it cannot yet be made a model case for rare disease research. Despite these limitations, this is a report to demonstrate the importance of research on rare diseases through research on Perry disease. It is expected that, in the future, other researchers will publish similar reviews for other rare diseases.

## 9. Conclusions

We reviewed our research topic, Perry disease, to reiterate the importance of research regarding rare diseases. Clinical and basic research regarding Perry disease has been described, including research history and collaboration. Rare diseases, like Perry disease, contribute to the study of common diseases. We hope that the research trajectory of Perry disease will serve as a model case for research on other rare neurodegenerative diseases. Far fewer researchers are involved in the study of rare diseases, so barriers to collaboration do exist. Nevertheless, there is collaboration among researchers, particularly clinicians and basic researchers; this is essential for research development. There is a need to establish an international collaboration system for solving these problems, and this may be the key to breakthroughs. The establishment of this system along with disease research requires support from governments and the industrial sector. Therefore, further development of industry–government–academia collaboration is proposed.

## Figures and Tables

**Table 1:** Diagnostic criteria for Perry disease.

Clinical Features		Laboratory Features
Cardinal	Supportive	Cardinal
(A) Parkinsonism *	(a) Rapid disease progression within five years of onset	(1) Genetic test: mutation in the *DCTN1* gene
(B) Apathy or depression	(b) Onset younger than 50 years	(2) Pathology: nigral neuronal loss and TDP-43 pathology in the brainstem and basal ganglia
(C) Respiratory symptoms **		
(D) Unexpected weight loss		
(E) Positive family history of parkinsonism or respiratory symptoms		
Definite: Presence of (A) and (E) plus cardinal laboratory features of (1) positive genetic test; Presence of (A), (B), (C), and (D) plus cardinal laboratory features of (1) positive genetic test; Presence of (A), (B), (C), and (D) plus cardinal laboratory features of (2) TDP-43 pathologies. If evidence of other mutations or neurodegenerative disease pathology is present, both cardinal laboratory features must also be observed.		
Probable: Presence of (A), (B), (C), (D), and (E).		
Possible: Presence of (A) and (E) plus supportive clinical features of (a) or (b).		

*: parkinsonism requires two or more among rigidity, tremor (with postural tremor acceptable), bradykinesia, and postural instability; **: respiratory symptoms require exclusion of cardiac and pulmonary diseases. Abbreviations: TDP-43, TAR DNA-binding protein 43.

**Table 2:** Comparison of clinical features of Perry disease, Parkinson’s disease, multiple system atrophy, progressive supranuclear palsy, and frontotemporal lobar degeneration.

Disease	Parkinsonism	Apathy/ Depression	Weight Loss	Respiratory Symptoms	Autonomic Dysfunction	Vertical Gaze Palsy
Perry disease	+	+	+	+	+	+
PD	+	+	+	-	+	-
MSA	+	+	±	+	+	-
PSP	+	+	-	-	-	+
FTLD	+	+	+	+	-	+

PD: Parkinson’s disease; MSA: multiple system atrophy; PSP: progressive supranuclear palsy; FTLD: frontotemporal lobar degeneration.

## Data Availability

No new data were created.
